# Evaluating Impulsivity as a Mechanism of Behaviour Change for 12-step Engagement during Alcohol Use Disorder Recovery

**DOI:** 10.21203/rs.3.rs-6680968/v1

**Published:** 2025-05-29

**Authors:** Emily E. Levitt, Kyla L. Belisario, Amanda Doggett, Allan Clifton, Robert Stout, John F. Kelly, James MacKillop

**Affiliations:** St. Joseph's Healthcare Hamilton; McMaster University; McMaster University; Vassar College; Pacific Institute for Research and Evaluation; Harvard Medical School; McMaster University

**Keywords:** Alcohol use disorder, Drinking, Impulsivity, Delay discounting, 12-step, Alcoholics Anonymous

## Abstract

**Objective:**

Participation in 12-step groups (TS), such as Alcoholics Anonymous, confers benefits among individuals with alcohol use disorder (AUD), and one candidate mechanism underlying these effects is reductions in impulsivity. Using a multidimensional assessment of impulsivity, the current study examined impulsive personality and action in a longitudinal cohort of adults with AUD initiating a significant recovery attempt.

**Methods:**

A prospective matched-sample cohort study design compared participants who reported a clinically meaningful increase in TS attendance (i.e., increase of ≥ 1 meetings/week; *n* = 74) from enrollment to 6 weeks to a matched control group who did not increase attendance (*n* = 74). Drinking was assessed using the Timeline Followback (% drinking days [%DD], % heavy drinking days [%HDD]); impulsivity was assessed using delay discounting (DD) and five UPPS-P subscales. Mediation models evaluated whether impulsivity explained the relationship between increased TS attendance and alcohol outcomes.

**Results:**

Compared to matched controls, participants who increased TS significantly differentially reduced %DD, %HDD (*p* < 0.001), as well as Negative Urgency (i.e., acting out due to negative emotions) and Lack of Perseverance (i.e., failure to persist in tasks; *ps* = 0.03) after their recovery attempt. Reductions in impulsivity, however, did not mediate the association between increased TS attendance and drinking.

**Conclusions:**

Increased TS attendance was associated with significant reductions in drinking and certain impulsivity traits. However, while the changes were contemporaneous, impulsivity did not explain the benefits of TS effects in early AUD recovery process. Future research should evaluate this hypothesis in larger samples and over longer follow-up periods.

## Public Health Significance Statement

While 12-step group participation is well-established as beneficial for reducing alcohol use, the specific mechanisms that explain its benefits remain unclear. Impulsivity is one candidate that has received relatively little attention. These findings reveal that certain aspects of impulsivity contemporaneously change with increased 12-step participation, but did not find evidence the impulsivity changes are causally responsible for 12-step benefits. Although further investigation is warranted, these findings do not suggest the benefits of 12-step are partially attributable to reductions in impulsivity.

## INTRODUCTION

Alcohol use disorder (AUD) is a major risk factor for global morbidity and mortality, with 3 million deaths annually linked to alcohol use (WHO, 2019). Twelve-step mutual-help organizations (MHOs), such as Alcoholics Anonymous (AA) are widely used, empirically-supported community-based resources for individuals seeking recovery from AUD (Alcoholics Anonymous, 2020; Hedden et al., 2015; Kelly et al., 2020). Studies consistently demonstrate the effectiveness of 12-step MHOs in initiating and sustaining recovery (Day et al., 2013; Lookatch et al., 2019; Litt et al., 2007), with a recent review suggesting that 12-step based interventions may outperform other well-established clinical interventions in improving recovery outcomes (Kelly et al., 2020).

Given the increased clarity regarding AA’s clinical and public health utility, there has been growing interest and research published regarding the mechanisms through which 12-step MHOs like AA confer relapse prevention benefit. This work has demonstrated that AA works through several psychological, social, and spiritual mechanisms (e.g., Humphreys et al, 1996; Kelly et al, 2009; Kelly et al, 2012; Kelly, 2017; Kelly et al, 2011; Tonigan et al, 2006). Despite strong evidence that AA mobilizes a variety of therapeutic mechanisms simultaneously to produce its salutary benefits (e.g., Kelly et al, 2012), only about half of the direct effect has been explained, thus far, through such multiple mechanisms as adaptive social network change (Kelly et al, 2011), boosting abstinence self-efficacy and relapse prevention coping skills, and increasing spirituality (Kelly et al, 2012). One further major mechanism of interest that has received little attention, however, is impulsivity.

Impulsivity has emerged as a promising candidate mechanisms of behaviour change in addiction recovery (Levitt et al., 2023) but has only been examined in a small number of studies in relation to 12-step (Blonigen et al., 2013; Kelly et al., 2018). Moreover, impulsivity is increasingly recognized as a multidimensional construct, comprising distinct facets such as impulsive decision-making (i.e., delay discounting [DD]) and impulsive personality traits (IPTs) (MacKillop et al., 2016). In the first case, DD is a behavioural economic index of impulsivity, reflecting a tendency to prefer smaller sooner rewards over larger delayed rewards, and greater DD indicates heightened impulsivity. In the second case, IPTs are typically assessed using the Barrett Impulsiveness Scales (Patton et al., 1995) or UPPS-P Impulsive Behaviour Scales (Whiteside & Lynam, 2001; Cyders et al., 2014). The UPPS-P captures five traits: Negative Urgency (acting impulsively in response to a negative state), Positive Urgency (acting impulsively in response to a positive state), Lack of Premeditation (acting without consideration of consequences), Lack of Perseverance (difficulty persisting with tasks), and Sensation Seeking (seeking novel and thrilling experiences). Elevated DD and IPTs have been consistently linked to AUD severity and risk of relapse (Amlung et al., 2017; Bernhardt et al., 2017; Coskunpinar et al., 2013) and reductions in these metrics predict better addiction treatment outcomes (Littlefield et al., 2015; Loree et al., 2015), making it a compelling candidate mechanism through which interventions like 12-step may confer benefit.

Twelve-step MHO participation may reduce impulsivity by promoting structure, goal-setting, and social accountability (Kelly et al., 2020; Blonigen et al., 2013). For example, attending meetings regularly and committing to recovery-related goals, may promote future-oriented thinking (e.g., “Thinking the drink through”) and AA’s strong emphasis on regular interpersonal consultation (e.g., with an AA sponsor) prior to making significant decisions reduces the tendency to act without foresight–both key components of impulsivity. It may also be that increased engagement with 12-step members allows for frequent observation of stable and contented sober peer role-models that can create optimism that a similarly contented positive “future self” is attainable for them too. This, in turn, can shift an individual’s decision-making towards valuing longer-term rewards, potentially reducing DD. Although these specific mechanisms have not been directly tested, preliminary evidence supports the idea that impulsivity may be implicated in the association between 12-step and recovery. For example, one prospective study of individuals with AUD found that longer 12-step participation was associated with greater reductions in impulsivity, which in turn partially mediated the associations between 12-step attendance and drinking outcomes 1 year later (Blonigen et al., 2013). This remains one of the few studies that explore impulsivity as a potential mechanism of behaviour change in 12-step, and while influential, it used a relatively uncommon and coarse measurement of impulsive traits. Further research using multifaceted, well-validated measures of impulsivity is needed to replicate and extend these findings.

The present study sought to explore whether impulsive decision making and IPTs represent mechanisms of behaviour change underlying the association between 12-step attendance and drinking outcomes. Participants were individuals with AUD initiating a substantial recovery attempt who completed assessments at baseline and 6 weeks later. This study had two primary aims: (1) to determine whether individuals who reported a clinically meaningful increase in 12-step attendance (i.e., attending at least one additional meeting per week following recovery initiation) experience improvements in drinking outcomes and impulsivity; and (2) to examine whether changes in impulsivity mediated the relationship between increased 12-step attendance and reduced alcohol use at 6 weeks, controlling for baseline variables.

## METHODS

### Study Design

Participants were part of a longitudinal study designed to examine the mechanisms of behaviour change in those with AUD making a significant recovery attempt. There were two study sites, one in Hamilton, Ontario, Canada, and one in Boston, Massachusetts, USA. Eligibility criteria were as follows: i) between 21-65 years old; ii) identify alcohol as their primary substance of choice; iii) meet diagnostic criteria for AUD; iv) report high risk drinking in the 30 days prior to their recovery attempt (defined as 7 or more drinks per week for females or 14 or more drinks per week for males); and v) have begun either formal AUD treatment or an informal recovery attempt within the past 90 days, or are planning to begin in the next 14 days. Participants were enrolled between May 2019 to September 2021, with a follow-up assessment after the recovery attempt occurring 6 weeks after enrollment. Recruitment originally took place at local inpatient and outpatient alcohol treatment centres, however, following the onset of lockdowns and restrictions related to COVID-19, participants were recruited from the public using online advertising strategies. Assessments were initially conducted through in-person interviews however they were adapted to a virtual format following the onset of COVID-19 pandemic. Ethics approval was obtained from the Hamilton Integrated Research Ethics Board (Protocol #3825) and the Partners Human Research Committee at Massachusetts General Hospital (Protocol #2017P002345).

To examine the impact of 12-step meeting attendance on drinking behaviours and impulsivity, a matched control group was extracted from the full study sample (*N* = 501). Participants who reported a clinically meaningful increase in meeting attendance (TS group; i.e., attending one or more meetings per week from baseline to 6 weeks) were demographically matched to participants who did not exhibit such an increase (control group). This approach was selected because only a small subset of the overall sample demonstrated a clinically meaningful increase in 12-step attendance, and this subgroup differed significantly from the full cohort. Propensity score matching was applied to reduce confounding and to approximate random assignment of participants (Rosenbaum & Rubin, 1983; Rubin, 1997).

### Measures

#### Drinking Outcomes

Drinking outcomes consisted of percent drinking days (%DD), percent heavy drinking days (%HDD; 4+/5+ drinks for females/males on a given occasion), and total drinks per week. All drinking outcomes at baseline were captured using the Timeline Follow-Back (TLFB; Sobell & Sobell, 1992), which is a calendar-based method of collecting daily consumption of standard alcoholic beverages for the past 90 days. At the 6-week follow-up, drinking outcomes for the past 45 days were captured by the TLFB, or, for those completing a virtual assessment, the Daily Drinking Questionnaire (DDQ; Collins, Parks, and Marlatt, 1985), which asks participants about average standard drinks consumed on each calendar day of the week.

#### Impulsivity Outcomes

Impulsive choice was measured by two 5-trial DD tasks of $100 and $1000 magnitudes. Both tasks aim to measure one’s preference for smaller, immediate monetary rewards relative to delayed but larger rewards, through the calculation of k-values, with larger values signifying preference for immediate rewards (Koffarnus and Bickel, 2014). A k-value was calculated for participants if they passed a quality control question of same monetary magnitude, whereby participants were instructed to choose which monetary value they would prefer with no time delay applied (i.e. correctly selecting the higher monetary value). The k-values were subsequently log (base-10) transformed as typically done in the field to create an approximately normal distribution (MacKillop et al., 2011).

Impulsive personality was measured by the UPPS-P, a 20-item questionnaire used to assess 5 factors of impulsivity: negative urgency, positive urgency, sensation seeking, lack of premeditation, and lack of perseverance. The four items from each factor were sum scored, with higher values signifying greater impulsivity (Cyders et al., 2014). Item-level missingness on the UPPS was handled using pro-rating by taking the mean score across the items for which they have data and multiplying it by the number of items in the instrument (Newman, 2014). Across the 5 factors at both baseline and follow-up, only a maximum of 1 item on a factor was missing for a very small sub-set of participants (0.00%–1.35%).

#### Twelve-Step Outcome

Twelve-Step attendance was calculated as the average number of meetings attended per week as captured by the TLFB or DDQ. These meetings included any 12-step meeting organization such as AA, Narcotics Anonymous, Cocaine Anonymous, Marijuana Anonymous, and Crystal Meth Anonymous. An increase in meetings at 6-weeks from baseline was considered clinically meaningful if participants increased their meeting attendance by 1 or more per week. Participants with a clinically meaningful increase in meeting attendance (TS group) were matched to controls who did not have a clinically meaningful increase, to artificially create a case-control design using a 1:1 optimal pair propensity score matching. Thus, for this purpose, those who had a clinically meaningful decrease in attendance (i.e. a decrease of 1 or more meetings per week) were excluded from the pool of control participants. Standardized mean differences less than ∣0.25∣ were considered balanced (Harder et al., 2010). Matching was done on: sex assigned at birth (female, male), race (racialized versus non-racialized), age, subjective household income to accommodate differences in average yearly earnings in Canada and the US (enough to pay bills versus not enough; Najdzionek et al., 2023), educational level (less than a bachelors degree versus a bachelors degree or higher), number of AUD symptoms at baseline, reported drinks per week at baseline, and endorsement of attendance of 12-step meetings (versus no attendance) at baseline. This ensured that participants were matched on demographics and drinking severity at baseline, minimizing differences between the case-control groups and allowing for the examination of the increase in 12-step meetings as a proxy for a randomly assigned treatment.

### Data Analysis

At baseline, 0.6% were excluded for responding to ≥3 quality control questions incorrectly, 3.4% for missingness on ≥3 quality control items, and 5.4% for missingness on demographic variables, 12-step meeting attendance, or impulsivity measures. At follow-up, retention was high (90.6%), but 6.2% were excluded for missing data or failed quality control. An additional 2.6% were excluded for having a substantial reduction in 12-step meeting attendance. See Supplemental Figure 1 for participant flow chart. Of the remaining sample (*n* = 380), *n* = 77 showed a clinically meaningful increase in 12-step meeting attendance at 6 week and were matched to *n* = 77 controls for a final sample of *n* = 154.

To quantify significant improvements within 12-step attendance from baseline to 6 weeks, linear mixed-effects models were conducted, relaxing the assumption that pre-recovery values between groups are the same (O’Connell et al., 2017). Significant time, or time-by-group effects were followed by post-hoc tests examining within- and between-group differences. Cohen’s d was calculated to assess the magnitude of change within-groups. Cross-sectional mediation models at 6 weeks were conducted using a structural equation modelling framework (SEM), allowing for auto-lagged effects of baseline measures and covariance between baseline measures. Significant impulsivity measures in the previous models were tested as mediators of the effects of 12-step attendance per week on drinking outcomes (%DD or %HDD).

Analyses were conducted using R (R Core Team, 2025). Matching was done with the *MatchIt* package (Ho et al., 2011), linear mixed-effects models and post-hoc tests with *lme4* (Bates et al., 2015), and *emmeans* (Lenth, 2025), and SEM models with *lavaan* (Rosseel, 2012).

## RESULTS

### Baseline Characteristics

The propensity score matching yielded balanced scores between groups, with an overall standardized mean difference of 0.23 (versus 1.03 prior to matching) on the eight baseline matched characteristics (see Supplemental Table S1 for matching characteristics at baseline). Adequate matching was confirmed through descriptive statistics (Table 1). There were no statistically significant differences between the TS group and matched control groups on drinking variables, 12-step attendance, or impulsivity measures at baseline.

### Changes at 6-Week Follow-Up

**Figure 1:**
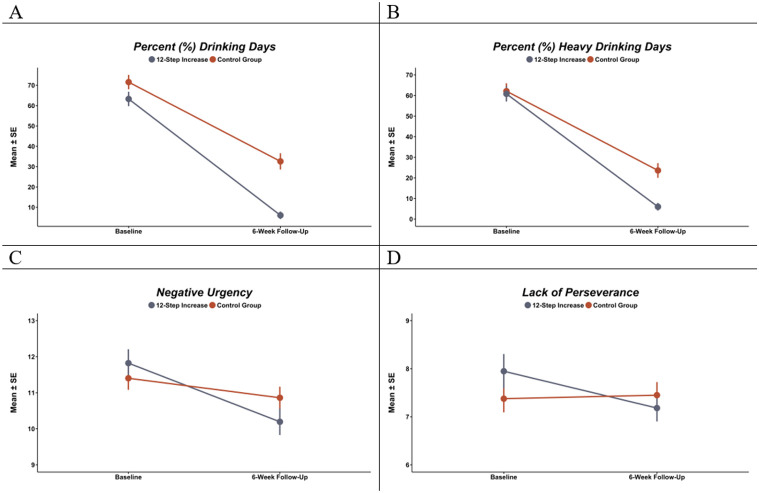
Baseline and 6-week follow-up mean and standard errors (SE) in: a) percent drinking days, b) percent heavy drinking days, c) negative urgency, and d) lack of perseverance. Statistically significant within-group differences denoted by above the 6-week SE bar, and significant between-group differences denoted below the bars. Note: * p < 0.05; ** p < 0.01; ** p < 0.005.

The TS group at 6 weeks attended more meetings per week (Mean ± SE = 4.7 ± 0.42) compared to controls (0.31 ± 0.09; *p* < 0.001). There were interaction effects of time with the TS group in the %DD and %HDD models, as reported in Table 2. The reduction in %DD was large in both groups (Figure 1), although larger in the TS group (Cohen’s d (95% CI) = 2.41 (2.03-2.78); *p* < 0.001) versus controls (1.61 (1.26-1.95); *p* < 0.001). Average %DD at 6 weeks was significantly lower for the TS group (Mean ± SE = 6.07 ± 1.87) compared to controls (32.40 ± 3.98; *p* < 0.001). The reduction in %HDD was larger in the TS group (*d* = 2.24 (1.87-2.61); *p* < 0.001) versus controls (*d* = 1.61 (1.27-1.96); *p* < 0.001), and %HDD was significantly lower in the TS group (5.99 ± 1.85) versus controls (23.25 ± 3.53; *p* < 0.001).

There was an interaction effect of time and TS group in the negative urgency models, with a significant reduction in negative urgency in the TS group (*d*(95%CI) = 0.73 (0.41-1.06); *p* < 0.001) but not controls (0.23 (−0.09-0.55); *p* = 0.152). There was a significant interaction effect of time with the TS group in the lack of perseverance models with a significant, moderate reduction in lack of perseverance in the TS group (0.48 (0.16-0.80); *p* = 0.003) but not controls (−0.02 (−0.34-0.30); *p* = 0.892). No other models had statistically significant effects.

### Mediational Analyses

In indirect effect testing, Negative Urgency did not exhibit a significant mediating effect on the relationship between number of 12-step meetings per week and %DD (*β* ± SE = −0.05 ± 0.10; *p* = 0.637) nor %HDD (−0.06 ± 0.07; *p* = 0.368). Similarly, there was no significant mediating effect of Lack of Perseverance on the relationship of number of 12-step meetings per week for either %DD (−0.04 ± 0.09; *p* = 0.653) or %HDD (−0.04 ± 0.06; *p* = 0.436).

## DISCUSSION

The current study examined whether facets of impulsivity, namely DD and IPTs, functioned as mechanisms of behaviour change in the relationship between 12-step attendance and alcohol use among individuals with AUD early in the recovery process. Specifically, this study examined whether individuals who reported a clinically meaningful increase in 12-step attendance experienced improvements in both drinking outcomes and impulsivity from baseline to 6 weeks post-recovery initiation, and whether changes in impulsivity mediated the relationship between increased 12-step attendance and reduced alcohol use at 6 weeks. Consistent with previous studies, a robust relationship was present between 12-step attendance and alcohol use. Individuals in the TS group reported over five times fewer drinking days and nearly four times fewer heavy drinking days than matched controls at 6 weeks. These findings align with a well-established literature supporting the effectiveness of 12-step MHO participation in reducing alcohol use (e.g., Kelly et al., 2020; Kaskuta, 2009). Notably, these effects remained even after controlling for baseline differences, highlighting the potential importance of engagement in 12-step MHOs during early recovery. Importantly, the TS group also showed significantly lower levels of negative urgency and lack of perseverance at 6 weeks. However, neither of these traits significantly mediated the association between 12-step attendance and reductions in drinking at follow-up. These findings are partially consistent with prior work by Blonigen et al. (2013), which found that increased 12-step attendance was associated with reductions in both drinking and impulsivity. In contrast to that study, however, which did find that a measure of impulsivity was a mediator of 12-step participation in relation to substance use outcomes, the current findings did not support impulsivity as a mediator of 12-step’s beneficial association with reduced alcohol use during the first 6 weeks of recovery. This inconsistency may be due to different sample characteristics, the different timepoints examined during the first year of recovery, and/or the different types of impulsivity measures used across the two studies.

The findings revealed significant group x time interactions for two impulsive personality traits: negative urgency and lack of perseverance. Compared to matched controls, the TS group showed greater reductions in these traits from baseline to 6 weeks, suggesting that increased 12-step attendance may be associated with early helpful changes in impulsivity during recovery. Although speculative, 12-step attendance may enhance emotion regulation and goal persistence, which are both conceptually linked to 12-step MHO principles and practices. For example, the structure and accountability provided by an AA sponsor and regular meeting attendance may enhance goal-directed behaviour, while the social and emotional support within the 12-step environment may reduce impulsive responses to negative affect.

In contrast, no significant interaction effects were observed for other IPTs (i.e., sensation seeking, lack of premeditation, positive urgency) or for DD. Several studies suggest that negative urgency and lack of premeditation are the IPTs most consistently linked to poor addiction treatment outcomes and are most likely to change over treatment (Hershberger et al., 2017). As such, the absence of change in lack of premeditation is somewhat unexpected and may reflect variability in how different facets of impulsivity respond to interventions. Conversely, the null findings for DD are consistent with current literature, suggesting that DD is relatively stable post-treatment and may reflect a trait-like construct, whereas IPTs may be more malleable and amenable to change (Littlefield et al., 2015; Levitt et al., 2023). Collectively, these findings emphasize the importance in distinguishing between facets of impulsivity when investigating mechanisms of recovery.

Despite promising group differences in IPTs, the mediation analyses did not support the hypothesis that reductions in impulsivity helped explain the link between increased 12-step attendance and reduced alcohol use. Several explanations may account for the lack of mediation. Impulsive personality traits may require more time to change than drinking behaviour, which can shift more quickly following intervention. Indeed, longitudinal work has suggested that changes in impulsivity are more pronounced later in recovery (Blonigen et al., 2013). Second, modest associations between variables and limited sample size may have reduced the power to detect mediation effects. It is plausible that mechanisms such as social network change, enhanced coping, or increased motivation and abstinence self-efficacy not tested in the current study may partially account for observed reductions in alcohol use, in keeping with the notion that recovery-related changes are dynamic and complex (e.g., Morgenstern et al, 1997; Kelly et al, 2012; Kelly, 2017). Additionally, increases in 12-step attendance and reductions in impulsivity occurred concurrently but were independent. Although speculative, it is possible both are linked to shared underlying factors, such as problem severity, readiness for change, or engagement with recovery goals–factors that were not measured in this study and warrant further investigation.

Any conclusions or generalizations from this study’s results should be made cautiously and only in light of several important limitations. First, although the overall sample size was relatively large, only a small portion of participants reported a clinically meaningful increase in 12-step attendance, potentially limiting power for the subgroup analyses. Second, while PSM helped balance participants on several baseline characteristics, this method cannot fully eliminate selection bias or support causal inference. Third, there was limited geographic and demographic diversity within the sample. The Canadian subsample was disproportionally large relative to the US subsample and attempts to match participants based on site revealed imbalance across several covariates. This may limit the generalizability of the findings, particularly in international populations or more diverse treatment settings.

In sum, this study adds further supporting evidence that 12-step attendance is associated with meaningful reductions in alcohol use and that increased 12-step attendance co-occurs with reductions in specific facets of impulsivity. An important nuance, however, is that these results do not support the hypothesis that the benefits of 12-step engagement are substantively determined by improvements in self-regulation, as measured by the impulsivity indices used. These findings highlight the dynamic complexity of behavioural and psychological change during the recovery process and suggest that the relationship between 12-step attendance and impulsivity may both be influenced by other factors in early recovery. Future research is needed to examine longer-term trajectories of change in impulsivity with larger and more diverse samples and explore a broader range of mechanisms that may explain how 12-step engagement promotes sustained recovery.

## Figures and Tables

**Table 1:** Baseline participant demographics (*N* = 152). Chi-square and t-tests examined significant baseline differences between 12-step increasers (↑TS) and matched controls.

Characteristic	Overall *N* = 154	TS *n* = 77	Controls *n* = 77	*p*
Demographics				
N (%) Study Site Location				0.10
Canada	93 (60.39%)	52 (67.53%)	41 (53.25%)	
USA	61 (39.61%)	25 (32.47%)	36 (46.75%)	
Mean ± SD Age	41.21 ± 11.11	41.10 ± 10.65	41.31 ±11.61	0.77
N (%) Female	75 (48.70%)	38 (49.35%)	37 (48.05%)	>0.90
N (%) Non-White	14 (9.09%)	8 (10.39%)	6 (7.79%)	0.78
N (%) 2SLGBTQIA+	33 (21.43%)	14 (18.18%)	19 (24.68%)	0.43
N (%) Subjective Household Income				>0.90
Not Enough for Bills	82 (53.25%)	41 (53.25%)	41 (53.25%)	
Enough for Bills	72 (46.75%)	36 (46.75%)	36 (46.75%)	
Median Subjective Household Income	Cut Back	Cut Back	Cut Back	0.67
N (%) Educational Attainment				0.61
< Bachelors Degree	100 (64.94%)	52 (67.53%)	48 (62.34%)	
Bachelors Degree or Higher	54 (35.06%)	25 (32.47%)	29 (37.66%)	
Median Educational Attainment	Associates Degree	Some Post-Secondary	Associates Degree	0.33
Alcohol-Related Measures				
Mean ± SE AUD Symptoms	9.42 ± 1.90	9.57 ± 1.98	9.27 ± 1.82	0.33
Mean ± SE Percent Drinking Days	67.43 ± 31.24	63.29 ± 31.16	71.58 ± 30.98	0.10
Mean ± SE Average Drinks per Week	60.40 ± 48.40	64.67 ± 50.47	56.13 ± 46.18	0.27
Mean ± SE Percent HDD	61.48 ± 32.66	60.81 ± 32.23	62.13 ± 33.27	0.80
12-Step-Related Measures				
N (%) Any 12-Step Meetings	56 (36.36%)	29 (37.66%)	27 (35.06%)	0.87
Mean ± SE 12-Step Meeting per Week	0.45 ± 1.02	0.51 ± 1.17	0.40 ± 0.86	0.48
Impulsivity-Related Measures				
Mean ± SE DD100 k-value (log)	−1.75 ± 0.68	−1.79 ± 0.74	−1.70 ± 0.63	0.44
Mean ± SE DD1000 k-value (log)	−2.09 ± 0.71	−2.09 ± 0.75	−2.09 ± 0.67	>0.90
Mean ± SE Sensation Seeking	10.46 ± 3.31	10.10 ± 3.13	10.82 ± 3.47	0.18
Mean ± SE Negative Urgency	11.61 ± 3.11	11.82 ± 3.38	11.40 ± 2.82	0.40
Mean ± SE Positive Urgency	9.29 ± 3.56	9.36 ± 3.66	9.22 ± 3.49	0.80
Mean ± SE Lack of Premeditation	8.16 ± 2.72	8.23 ± 2.80	8.10 ± 2.64	0.75
Mean ± SE Lack of Perseverance	7.66 ± 2.84	7.95 ± 3.13	7.38 ± 2.50	0.21

**Table 2:** Omnibus statistics for the main and interaction effects of the linear mixed effects models. Significant effects (*p*<.05) are in boldface.

Outcome	Time		TS	Time × Group		
	*F*	*p*	*F*	*p*	*F*	*p*
	*Drinking Outcomes*					
% Drinking Days	**99.43**	**0.001**	**19.45**	**0.001**	**12.31**	**0.001**
% Heavy Drinking Days	**100.39**	**0.001**	**6.65**	**0.011**	**7.50**	**0.007**
	*Impulsivity Measures*					
DD100 k-value (log)	0.76	0.38	1.80	0.18	0.29	0.59
DD1000 k-value (log)	0.29	0.59	0.64	0.43	0.78	0.38
Sensation Seeking	1.29	0.26	2.10	0.15	0.04	0.83
Negative Urgency	2.08	0.15	0.002	0.96	**4.85**	**0.03**
Positive Urgency	1.72	0.19	0.17	0.68	0.16	0.69
Lack of Premeditation	0.21	0.65	0.46	0.50	1.56	0.21
Lack of Perseverance	0.02	0.89	0.22	0.64	**4.91**	**0.03**

**Table 3:** Unstandardized beta coefficients (standard errors) from the mediation models pathways, as well as indirect and total effects of drinking measure on impulsivity.

% DD	Meetings to Impulsivity (a)	Impulsivity to % Days (b)	Meetings to % Days (c’)	Indirect Effect (a*b)	Total Effect (a*b + c’)
Negative Urgency	−0.13 (0.06); **p = 0.028**	0.35 (0.73); p = 0.629	−2.80 (0.64); **p < 0.001**	−0.05 (0.10); p = 0.637	−2.85; **p < 0.001**
Lack of Perseverance	−0.13 (0.06); **p = 0.029**	0.31 (0.67); p = 0.646	−2.16 (0.57); **p < 0.001**	−0.04 (0.09); p = 0.653	−2.20 (0.57); **p < 0.001**
% HDD	Meetings to Impulsivity (a)	Impulsivity to % HDD (b)	Meetings to % HDD (c’)	Indirect Effect (a*b)	Total Effect (a*b + c’)
Negative Urgency	−0.06 (0.04); p = 0.194	1.09 (0.88); p = 0.212	−2.86 (0.63); **p < 0.001**	−0.06 (0.07); p = 0.368	−2.92 (0.63); **p < 0.001**
Lack of Perseverance	−0.06 (0.04); p = 0.196	0.78 (0.80); p = 0.329	−2.20 (0.57); **p < 0.001**	−0.04 (0.06); p = 0.436	−2.25 (0.57); **p < 0.001**
